# On the Influence of the Last Inundation on the Health and Population of Lyons

**Published:** 1842-03

**Authors:** 


*On the Influence of the last Inundation on the health of the Population of Lyons.*—It was a matter of much interest to determine what were the effects on the health of its inhabitants of the inundation, which, in so short a time, on two different occasions, covered a part of the town of Lyons. If mere variations of temperature and atmospheric moisture do exercise on the health only a part of the fatal effects attributed to them, it is certainly, in a case like that of Lyons, that such effects ought to be easily appreciable. In no inundation of that town had the waters ever before risen to such a height: in none was their stay so long, or their retreat so slow. They covered the town and the country round over an area of several leagues; they inundated the quays, the streets, the places of public resort, the cellars, the houses; and they polluted the waters of the wells, the pumps, and springs, with heterogeneous substances that filtered into them from the sewers and the privies. After their retreat a stinking sediment covered the majority of the streets, and a thick viscid mould lined the inner walls of the houses; the atmosphere was charged with a nauseous dampness; the population was distressed; everything was dreary; and yet the inundation of 1840 had but a just perceptible influence upon the public health. From the month of November in that year, to the present time (July, 1841), the hospitals have not been more than usually encumbered: the number of patients has been but little increased, and, though the mortality has been distinctly greater, yet this cannot be attributed to any disease in particular produced by the inundation.

In the days immediately following this scourge, a considerable number of cases of obstinate diarrhœa, and some of dysentery attributed to drinking the unwholesome water, were observed. Some typhoid fevers also appeared; and, if the state of the atmosphere had any influence upon them, it was only by prolonging their continuance, and giving them a more marked adynamic character. Still they were not more fatal than they usually are, though it was necessary to modify the ordinary treatment; blood-lettings at the outset were less employed, and tonics were sooner and longer administered.

At a later period numerous and obstinate rheumatisms were observed, but they rarely had an acute character, except in those who had worked long in the water: in general the moisture of the atmosphere rather revived old pains than generated new ones. The diseases which reigned most generally, were catarrhs and catarrhal fevers, which, however, are habitually prevalent at Lyons at this season; but the inundation remarkably increased their number and modified their character; some affecting the lungs, had an alarming intensity and obstinacy, but on the whole they destroyed but few. An epidemic disease, of which both the etymology and etiology are still obscure, reigned at this time at the garrison, but it could not be, in any measure, traced to the effects of the inundation.

Notwithstanding the absence of any special affection, however, the mortality was more considerable than during the preceding years. Thus, in the last two months of 1840, the town and hospitals together had 1118 deaths; while in the same two months of 1839, there were but 787, and of 1838 only 750. But this difference is easily explained by the pernicious influence such a scourge would have on aged, weak, and diseased people, and especially on such as were already affected with chronic diseases.

The author attributes the good state of health of the town and its environs, during the continuance of the scourge, to the prompt and energetic measures which were taken by the local administration, and to the north wind, which continued for twenty days and greatly aided the sanitary measures that were employed.—*Lond. Med. Gaz. November* 5, 1841, from *Journal de Medecine de Lyons*, Juillet, 1841.

